# Mitochondrial DNA with a Large-Scale Deletion Causes Two Distinct Mitochondrial Disease Phenotypes in Mice

**DOI:** 10.1534/g3.113.007245

**Published:** 2013-09-01

**Authors:** Shun Katada, Takayuki Mito, Emi Ogasawara, Jun-Ichi Hayashi, Kazuto Nakada

**Affiliations:** * Graduate School of Life and Environmental Sciences, University of Tsukuba, 1-1-1 Tennoudai, Tsukuba, Ibaraki 305-8575, Japan; † Faculty of Life and Environmental Sciences, University of Tsukuba, 1-1-1 Tennoudai, Tsukuba, Ibaraki 305-8575, Japan; ‡ International Institute for Integrative Sleep Medicine (WPI-IIIS), University of Tsukuba, 1-1-1 Tennoudai, Tsukuba, Ibaraki 305-8575, Japan

**Keywords:** mitochondria, mitochondrial DNA, pathogenic mutation, mitochondrial diseases, model mice

## Abstract

Studies in patients have suggested that the clinical phenotypes of some mitochondrial diseases might transit from one disease to another (*e.g.*, Pearson syndrome [PS] to Kearns-Sayre syndrome) in single individuals carrying mitochondrial (mt) DNA with a common deletion (∆mtDNA), but there is no direct experimental evidence for this. To determine whether ∆mtDNA has the pathologic potential to induce multiple mitochondrial disease phenotypes, we used *trans*-mitochondrial mice with a heteroplasmic state of wild-type mtDNA and ∆mtDNA (mito-mice∆). Late-stage embryos carrying ≥50% ∆mtDNA showed abnormal hematopoiesis and iron metabolism in livers that were partly similar to PS (PS-like phenotypes), although they did not express sideroblastic anemia that is a typical symptom of PS. More than half of the neonates with PS-like phenotypes died by 1 month after birth, whereas the rest showed a decrease of ∆mtDNA load in the affected tissues, peripheral blood and liver, and they recovered from PS-like phenotypes. The proportion of ∆mtDNA in various tissues of the surviving mito-mice∆ increased with time, and Kearns-Sayre syndrome−like phenotypes were expressed when the proportion of ∆mtDNA in various tissues reached >70–80%. Our model mouse study clearly showed that a single ∆mtDNA was responsible for at least two distinct disease phenotypes at different ages and suggested that the level and dynamics of ∆mtDNA load in affected tissues would be important for the onset and transition of mitochondrial disease phenotypes in mice.

Pathogenic mtDNAs having a large-scale deletion (∆mtDNA), called the “common deletion,” or a point mutation, induce defects of mitochondrial oxidative phosphorylation (mitochondrial respiration defects) and manifest as a wide variety of mitochondrial diseases (Larsson and Clayton 1995; Wallace 1999). It has been well documented that ∆mtDNA is responsible for three clinical phenotypes of mitochondrial diseases: Kearns–Sayre syndrome (KSS), progressive external ophthalmoplegia, and Pearson syndrome (PS) (DiMauro and Garone 2011). KSS is usually sporadic and is characterized by early onset (<20 years of age), lactic acidosis, chronic progressive external ophthalmoplegia, pigmentary retinopathy, heart block, diabetes, deafness, cerebellar abnormalities, and renal failure. Progressive external ophthalmoplegia is mainly a skeletal muscle disorder and is characterized by late-onset progressive external ophthalmoplegia, lactic acidosis, myopathy, and exercise intolerance. PS is a rare disorder of early infancy that is characterized mainly by sideroblastic anemia. At present, the precise mechanism by which ∆mtDNA can cause different disease phenotypes is unclear, although it has been considered that different loads of ∆mtDNA in tissues are important to establish the multiple disease phenotypes.

Some studies in patients have reported the possibility that infants with PS who survive into childhood develop the clinical features of KSS (Larsson *et al.* 1990; McShane *et al.* 1991; Rotig *et al.* 1995). Furthermore, Larsson *et al.* (1990) suggest that the transition of disease phenotypes is governed by the fractional concentration of ∆mtDNA in various tissues; however, there is currently no experimental evidence for this. Because mitochondrial function is regulated both by nuclear DNA and mtDNA (Larsson and Clayton 1995), it is possible that nuclear genomic background is involved in the pathogenesis of different disease phenotypes. Supporting this hypothesis is the finding that the homoplasmic state of a pathogenic mutant mtDNA does not always induce serious clinical phenotypes in a mother and her children (McFarland *et al.* 2002).

Here, to experimentally resolve the question of whether mitochondrial disease phenotypes can change in single individuals as a result of the dynamics of the proportion of ∆mtDNA in various tissues, we have used a mouse model carrying pathogenic ∆mtDNA. Previously, we succeeded in generating a *trans*-mitochondrial mouse model (mito-mice∆) with a heteroplasmic state for wild-type mtDNA and ∆mtDNA with a 4696-bp deletion from nucleotide position 7759 of the *tRNA^Lys^* gene to position 12,454 of the *ND5* gene (see Figure 1A) (Inoue *et al.* 2000). The ∆mtDNA introduced in mito-mice∆ is similar to the pathogenic mutant mtDNA with the “common deletion” that is found in patients with KSS or PS (Holt *et al.* 1988; Larsson *et al.* 1990; McShane *et al.* 1991; Rotig *et al.* 1995). Because the mito-mice∆ all share the same nuclear genomic background (C57BL/6, also called B6), their genetic variation is restricted to the load of ∆mtDNA in various tissues. The mito-mice∆, therefore, would provide the direct experiment evidence whether a single ∆mtDNA could cause the onset and transition of different mitochondrial disease phenotypes.

**Figure 1:**
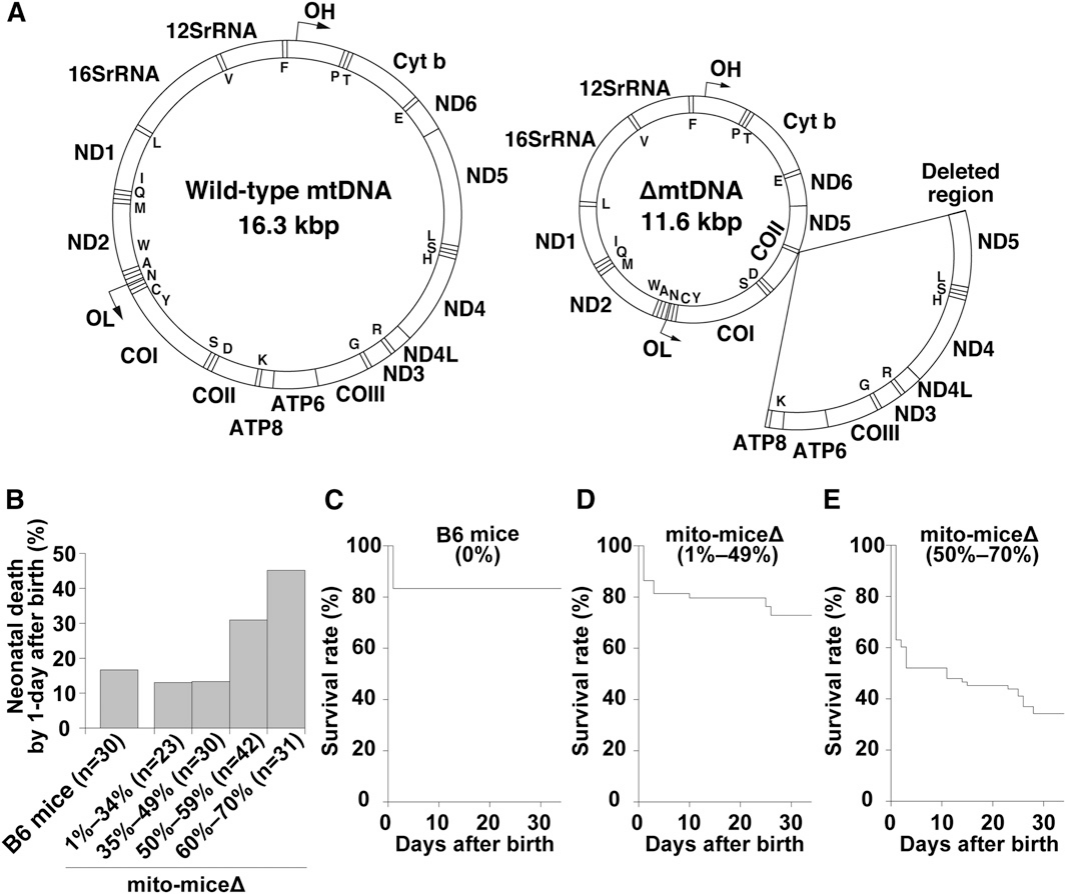
Gene map of ∆mtDNA introduced in mito-mice∆ and observations of mito-mice∆ on the early life. (**A**) A wild-type mtDNA (left) is a circular DNA (16.3 kbp) and encodes 37 genes, including 13 mRNAs, 22 tRNAs, and 2 rRNAs. A mtDNA with a common deletion (∆mtDNA; right) introduced in mito-mice∆ is 11.3 kbp in size, because 13 genes, including 7 mRNAs and 6 tRNAs (arc) are lost by a deletion. Single capitals in maps indicate tRNA genes. (B) Frequency of neonatal death in mito-mice∆ carrying 1–70% ∆mtDNA in their tails. Neonates of mito-mice∆ were classified into four groups carrying 1–34% (*n* = 23), 35–49% (*n* = 30), 50–59% (*n* = 42), and 60–70% (*n* = 31) ∆mtDNA, respectively, in their tails. Neonates of wild-type B6 mice were used as normal controls (*n* = 30). (C–E) Life span of mito-mice∆ carrying 1–70% ∆mtDNA in their tails was assessed for days 1 to 34. Based on the observed frequency of neonatal death in mito-mice∆ (see panel B), neonates of mito-mice∆ were classified into two groups carrying 1–49% (*n* = 53) (D) and 50–70% (*n* = 73) (E) ∆mtDNA in their tails, and the life span of each group was examined. The life span of wild-type B6 mice was also examined as a normal control (*n* = 30) (C). Survival curves were compared between mito-mice∆ carrying 1–49% ∆mtDNA and wild-type B6 mice (*P* = 0.3054, log-rank test) and between mito-mice∆ carrying 50–70% ∆mtDNA and wild-type B6 mice (*P* < 0.0001, log-rank test).

By using these mito-mice∆, we investigated that ∆mtDNA possessed a pathogenic potential for the onset of two distinct phenotypes of mitochondrial diseases, PS-like and KSS-like phenotypes, early in life (days 1−34 after birth) and in midlife (6 months of age), respectively. We also observed that the mice that escaped from the early death probably because PS-like phenotypes developed KSS-like phenotypes in middle age, showing the transition of distinct mitochondrial disease phenotypes in single mito-mice∆.

## Materials and Methods

### Mice

Mito-mice∆ were generated by introducing ∆mtDNA from cultivated cells into the zygotes of B6 strain mice by using cell fusion techniques, as described previously (Inoue *et al.* 2000). Thirty-three female mito-mice∆ carrying 1–63% ∆mtDNA at 2–6 months of age were used as mothers. Embryos on E18.5 and progeny were used for the study. Age-matched wild-type B6 mice were used as normal controls. All animal experiments were performed in compliance with the institutional guidelines of the University of Tsukuba for the care and use of laboratory animals.

### Estimation of ΔmtDNA proportion in tissues

Proportions of wild-type mtDNA and ∆mtDNA were determined by using real-time polymerase chain reaction, as described previously (Sato *et al.* 2005). Tail samples were used to deduce the ∆mtDNA load in mito-mice∆. The proportion of ∆mtDNA was also examined in various tissue samples (peripheral blood, liver, kidney, and eye).

### Measurement of blood lactate concentration

For the measurement of blood lactate concentration, blood was collected from the tail vein of nonfasted mice. Lactate concentration was measured by using an automatic blood lactate test meter (Lactate Pro, ARKRAY, Kyoto, Japan).

### Cytologic and histologic analysis

Blood samples were smeared on glass slides, and inclusions in reticulocytes were visualized by staining with new methylene blue. Cells with granular network inclusions that are a typical structure of reticulocytes were counted (see open arrowheads in Figure 3D) and proportion of these reticulocytes to 1000 red blood cells was estimated. The proportion of reticulocytes in blood samples of mito-mice∆ and age-matched controls was measured. To detect the accumulation of ferric iron, paraffin-embedded sections (5-μm thick) of liver samples were stained with Prussian blue and then Safranin O was used as a counter-staining. Eye samples were fixed in Bouin’s solution, and paraffin-embedded sections (8-μm thick) of the samples were stained with hematoxylin and eosin.

### Electron microscopic observation of cytochrome *c* oxidase (COX) activity

An electron microscopic analysis of COX activity was performed as described previously (Nakada *et al.* 2001; Seligman *et al.* 1968) with slight modifications. To summarize, 25-μm cryosections of liver samples were fixed in 2% w/v glutaraldehyde in phosphate-buffered saline for 5 min at 0°. Ultrathin sections, which were not stained with uranyl acetate or lead nitrate solutions, were viewed directly on a transmission electron microscope (model H-7650, Hitachi High-Technologies Corporation, Tokyo, Japan). In this analysis, enzymatic activity of COX was visualized as a black color (high electron density).

### Statistics

The Student’s *t*-test was used to compare groups of data. All values are presented as means ± SD. The log-rank test was used to compare survival curves. *P* < 0.05 was considered to indicate statistical significance.

## Results

### Frequency of early death in mito-miceΔ

We first examined the early life spans (1–34 days after birth) of mito-mice∆ carrying 1–70% ∆mtDNA in their tails (*n* = 126). The proportion of ∆mtDNA in individual mito-mice∆ was deduced from that in their tails just after birth (day 0). In this assay, we also used wild-type B6 mice as a normal control (*n* = 30). Approximately 20% of wild-type B6 neonates died on day 1 after birth (Figure 1B), but none died from days 2 to 34 (Figure 1C). The early life spans of mito-mice∆ carrying <50% ∆mtDNA in their tails were similar to those of wild-type B6 mice (Figure 1, C and D). In contrast, when the ∆mtDNA load in the neonates’ tails was ≥50%, the frequency of death on day 1 after birth was definitely increased compared with that of wild-type B6 mice (Figure 1B). The mito-mice∆ carrying 50–70% ∆mtDNA in their tails continued to die after day 1, and only 34% of these neonates survived until day 34 after birth (Figure 1E).

**Figure 2:**
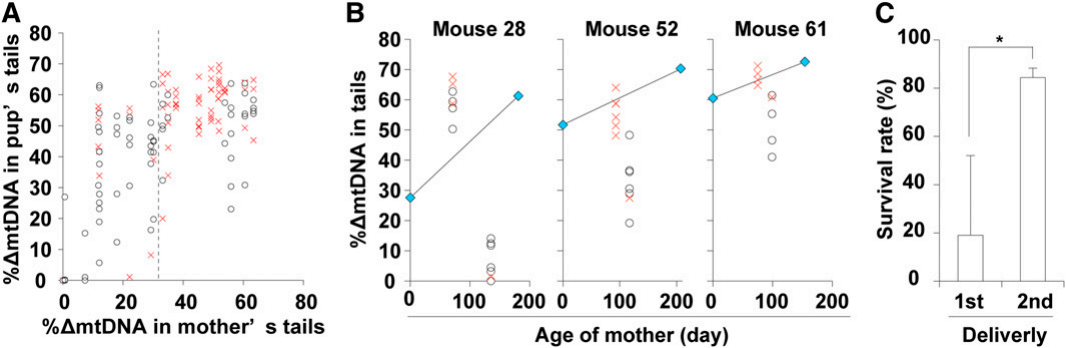
Relationship between early death and ∆mtDNA load in mito-mice∆. (A) Comparison of ∆mtDNA proportion in tail samples from pups and mothers. Open circles and red crosses indicate pups that were alive and dead, respectively, at 34 days after birth. (B) Comparison between first and second litters in terms of pup survival and ∆mtDNA proportion in the tails of pups. Three female mito-mice∆ carrying 28% (Mouse 28), 52% (Mouse 52), and 61% (Mouse 61) ∆mtDNA in their tails just after birth (day 0) were used in this assay. Open circles and red crosses indicate the proportions of ∆mtDNA in the tails of pups that were alive and dead, respectively, at 34 days after birth. Blue symbols indicate the proportions of ∆mtDNA in mother’s tails. (C) Comparison between the first and second deliveries in terms of survival rates of pups from Mouse 28, Mouse 52, and Mouse 61 at day 34 after birth. Data are presented as mean ± SD. Asterisk indicates significant differences (*P* < 0.05).

Although the aforementioned results indicated that most mito-mice∆ carrying ≥50% ∆mtDNA died early (≤34 days after birth), a substantial proportion of the progeny of mothers carrying >33% ∆mtDNA died very early (day 1 after birth; Figure 2A). Thus, it was unclear that early death was associated with the proportion of ∆mtDNA either in neonates or mothers. If the latter were the case, then the frequency of early death of pups would be expected to increase with the age of the mother, because ∆mtDNA accumulates in various somatic tissues but disappears in eggs with time (Sato *et al.* 2007). Therefore, we next examined the frequency of early death in the first and second litters of three female mito-mice∆ carrying 28% (Mouse 28), 52% (Mouse 52), and 61% (Mouse 61) ∆mtDNA, respectively, in their tails just after birth. As expected, the proportion of ∆mtDNA was much greater in the first litter than the second litter (Figure 2B). The rate of early death of pups was greatly and significantly greater in the first litter than in the second delivery (Figure 2, B and C; *P* < 0.05). The proportion of surviving pups from the second litter (Figure 2C) was comparable with that of pups with wild-type B6 mothers (Figure 1B), despite the ∆mtDNA load of the mothers’ tails increasing with time. Average loads of ∆mtDNA in tail samples were 32.4 ± 21.9% (mean ± SD) and 57.4 ± 16.8% in live and dead mito-mice∆ pups, respectively. On the basis of these results, we concluded that early death of mito-mice∆ was caused by ∆mtDNA load in neonates but not in mothers.

### Phenotypic observations of late-stage embryos of mito-miceΔ

**Figure 3:**
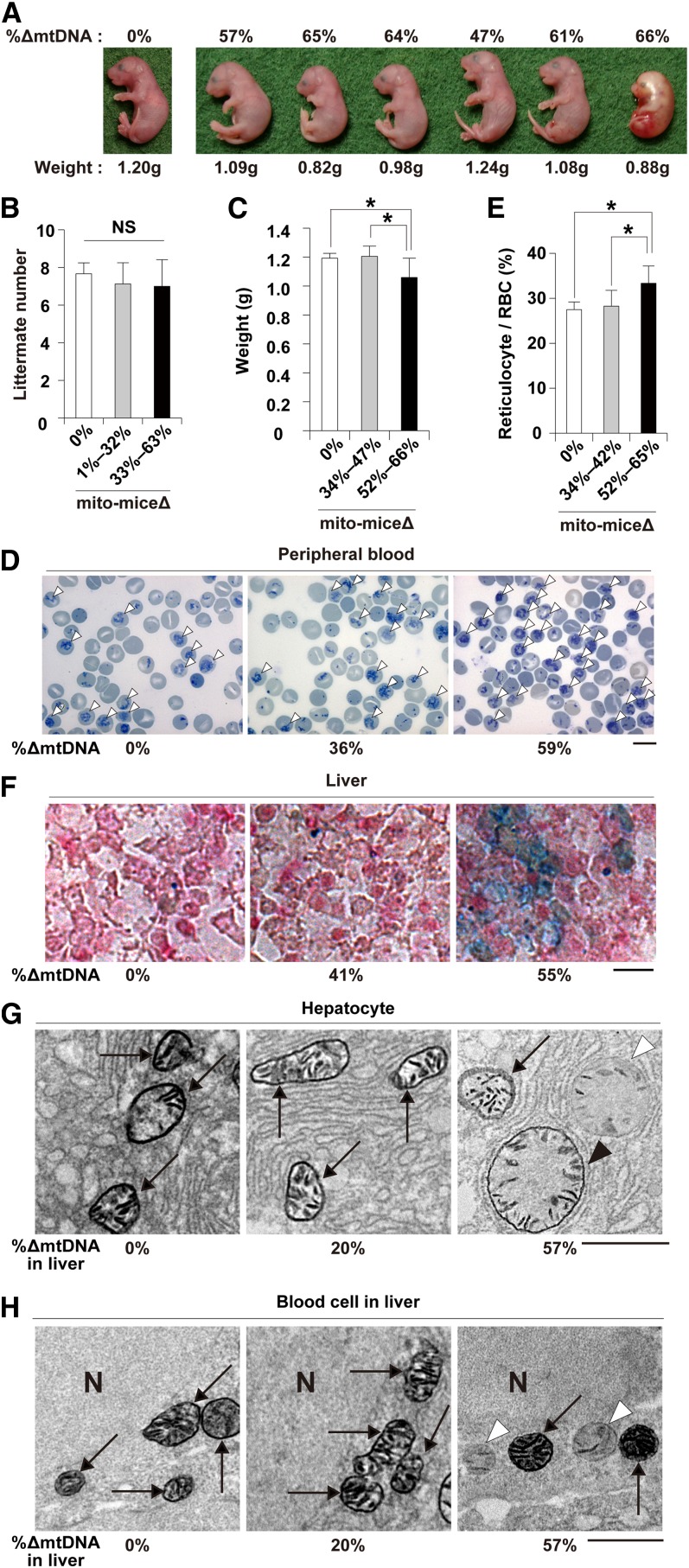
Phenotypic observations of late-stage embryos of mito-mice∆. All embryos were obtained at embryonic day 18.5. (A) Morphological observation of late-stage embryos carrying 0–66% ∆mtDNA in their tails, as indicated. The embryo carrying no ∆mtDNA (left side) was a wild-type B6 control. The six embryos carrying ∆mtDNA were obtained from a single mother. The weight of each embryo is shown below the panel. (B) Comparison of littermate number. Average numbers of littermates from mothers carrying 0% (white), 1–32% (gray), or 33–63% (black) ∆mtDNA. Data are presented as mean ± SD. NS, not significant. (C) Relationship between weight and ∆mtDNA load in late-stage embryos carrying 0% (white), 34–47% (gray), or 52–66% (black) ∆mtDNA. Data are presented as mean ± SD. Asterisks indicate significant differences (*P* < 0.05). (D) Cytological observation of peripheral blood samples from late-stage embryos. Peripheral blood samples carrying 0%, 36%, and 59% ∆mtDNA, respectively, were stained with new methylene blue. In this method, reticulocytes, a type of immature red blood cells (RBC), are visualized as cells with blue inclusions (open arrowheads). Scale bar, 10 μm. (E) Average proportion of reticulocytes in total RBCs in peripheral blood samples from late-stage embryos carrying 0% (white), 34–42% (gray), or 52–65% (black) ∆mtDNA. Data are presented as mean ± SD. Asterisks indicate significant differences (*P* < 0.05). (F) Histologic observations of iron metabolism in liver samples from late-stage embryos. Liver samples carrying 0%, 41%, and 55% ∆mtDNA, respectively, were stained with Prussian blue. In this method, abnormal iron metabolism is visualized as blue deposits. Scale bar, 10 μm. (G) and (H) Electron microscopic observation of COX activity in liver samples from late-stage embryos. Mitochondria in hepatocytes and blood cells are shown as (G) and (H), respectively. Arrows indicate mitochondria that appeared normal in shape and COX activity, open arrowheads indicate COX-deficient mitochondria, and the closed arrowheads indicate swollen mitochondria with COX activity. N, nucleus. Scale bar, 1 μm.

To clarify the reason for the frequent early death in progeny carrying ≥50% ∆mtDNA in their tails, we examined clinical phenotypes in embryos at embryonic day 18.5 (E18.5), which is just 1 day before birth. We obtained 55 E18.5 embryos carrying 35–70% ∆mtDNA in their tails. We also examined 55 E18.5 embryos from wild-type B6 mice as a normal control. None of the E18.5 embryos of wild-type B6 mice showed lethal phenotypes. Among the E18.5 embryos from mito-mice∆, three (5.5%) showed lethal phenotypes, and their tails carried 66%, 67%, and 70% ∆mtDNA, respectively, indicating that embryonic lethal phenotypes were restricted to embryos carrying a high load of ∆mtDNA. A typical example of an embryo with a lethal phenotype is shown in Figure 3A (right side): the embryo was atrophied and discolored when compared with littermate embryos carrying <66% ∆mtDNA in their tails. However, there was no significant difference in littermate number between normal mice and mito-mice∆ at E18.5 (Figure 3B). Thus, we concluded that the embryonic lethality was a rare event that was difficult to relate to the observation of frequent early death in mito-mice∆.

We then examined the relationship between body weight and ∆mtDNA load in tails (Figure 3, A and C). The average body weight of wild-type E18.5 embryos was 1.19 ± 0.03 g (*n* = 7), and that of E18.5 embryos carrying 34–47% ∆mtDNA (1.20 ± 0.07 g, *n* = 8) was in the normal range. In contrast, a substantially lower body weight was seen in E18.5 embryos carrying 52–66% ∆mtDNA (1.05 ± 0.13 g, *n* = 14, *P* < 0.05). Thus, growth retardation was induced preferentially in E18.5 embryos carrying ≥50% ∆mtDNA loads in tails, which was associated with frequent early death after birth. It is well known that ∆mtDNA is a genetic candidate for involvement in the pathogenesis of PS, which is a rapidly fatal disorder of infancy characterized mainly by sideroblastic anemia (DiMauro and Garone. 2011). Our results suggested the possibility that neonates carrying ≥50% ∆mtDNA may show clinical phenotypes of PS. We therefore examined whether E18.5 embryos carrying ≥50% ∆mtDNA showed sideroblastic anemia. Typical sideroblasts were not observed in peripheral blood samples from E18.5 embryos carrying ≥50% ∆mtDNA. In contrast, compared with normal E18.5 embryos, the number of reticulocytes in peripheral blood samples from E18.5 embryos carrying ≥52% ∆mtDNA showed a statistically significant increase (Figure 3, D and E; *P* < 0.05), although the increase was slight. The proportion of reticulocytes in peripheral blood samples from E18.5 embryos carrying ≤42% ∆mtDNA was similar to that in samples from normal E18.5 embryos (Figure 3, D and E). These data suggested abnormalities of hematopoiesis and the resultant onset of mild anemia in E18.5 embryos carrying ≥50% ∆mtDNA. Because abnormal iron metabolism in liver is observed in cases of sideroblastic anemia and PS (Camaschella. 2008; Gurgey *et al.* 1996), we examined pathologic changes in liver samples from E18.5 embryos carrying ∆mtDNA. Deposits of ferric iron indicating abnormal iron metabolism were preferentially observed in liver samples from E18.5 embryos with an increased number of reticulocytes (Figure 3F). On the basis of these observations, we concluded that ≥50% ∆mtDNA loads could cause PS-like phenotypes in late-stage mouse embryos, although it was not a typical PS.

In our previous studies using mito-mice∆, accumulation of approximately 70–80% ∆mtDNA loads in various tissues was necessary to induce mitochondrial respiration defects, such as deficiencies of complex IV (COX) in mitochondrial respiratory chains, and the resultant onset of various clinical phenotypes. In contrast, E18.5 embryos showed clinical phenotypes, even when they carried ≥50% ∆mtDNA. These results were expected that threshold for tolerance to accumulation of ∆mtDNA in embryonic liver and blood cells would be lower than that in other tissues. Because electron microscopic observations of COX activity (COX-EM) can visualize COX activity in individual mitochondria of single cells (Nakada *et al.* 2001; Seligman *et al.* 1968), we next performed COX-EM with liver samples from E18.5 embryos carrying various loads of ∆mtDNA. In liver samples carrying ≥50% ∆mtDNA, we observed three types of mitochondria in a single cytoplasm of hepatocytes, mitochondrion that appeared normal in COX activity (arrows in Figure 3G), a swollen mitochondrion with COX activity (closed arrowhead in Figure 3G), and a COX-deficient swollen mitochondrion (open arrowhead in Figure 3G). In blood cells of liver samples carrying ≥50% ∆mtDNA, heterogeneity of COX activity within individual mitochondria was clearly visible (Figure 3H), although swollen mitochondria were not observed. These observations indicated that ≥50% ∆mtDNA loads could induce mitochondrial respiration defects in hepatocytes and blood cells of late-stage embryos. In addition, there were no autophagic ultra-structures around abnormal mitochondria in hepatocytes or blood cells (Figure 3, G and H), indicating that that the COX-deficient mitochondria were not eliminated by the mitophagy, even when a single cytoplasm contained mitochondria with different mitochondrial respiratory functions.

### Changes of ΔmtDNA load and clinical phenotypes in mito-miceΔ during early life

**Figure 4:**
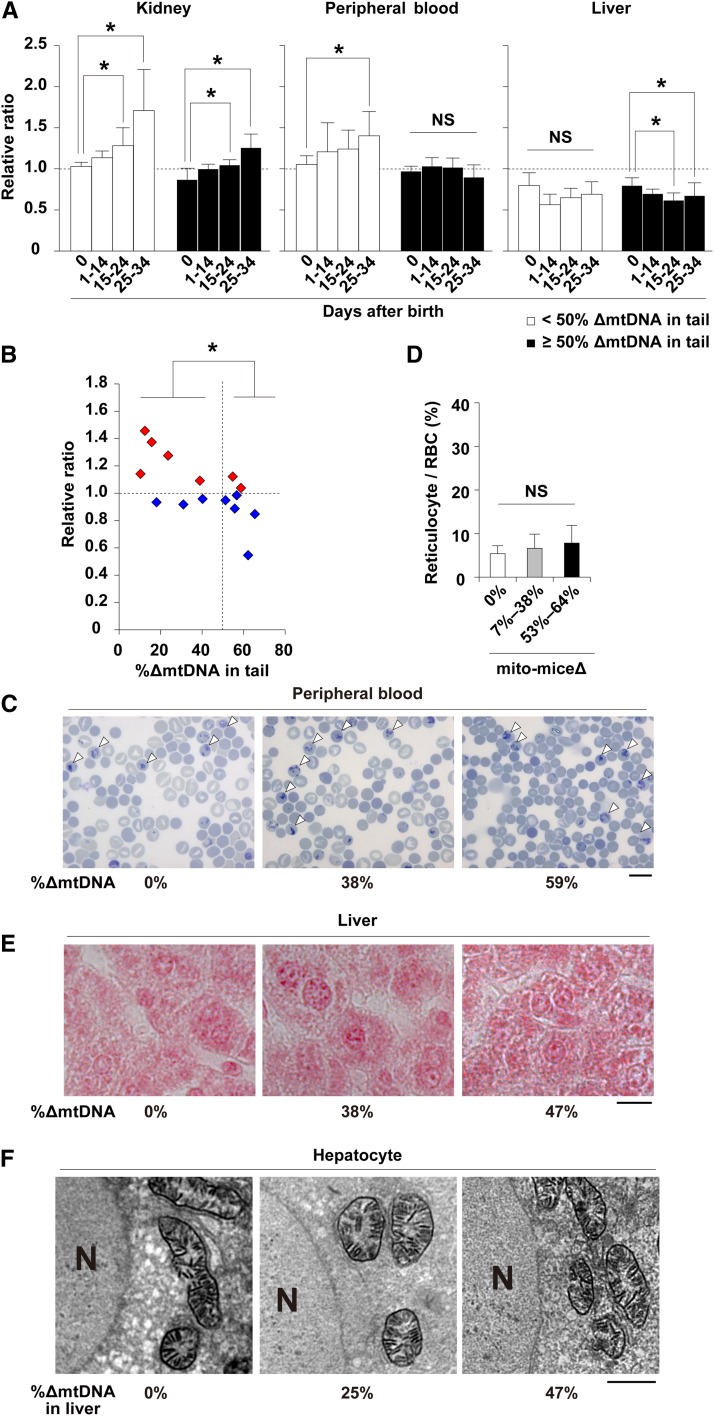
Dynamics of the proportion of ∆mtDNA in early life and phenotypic observations of mito-mice∆. (A) Dynamics of proportion of ∆mtDNA in affected (peripheral blood and liver) and nonaffected (kidney) tissues at various time points up to and including day 34 after birth. White and black bars indicate populations of mito-mice∆ carrying <50% and ≥50% ∆mtDNA in tail samples, respectively. The relative ratio is the proportion of ∆mtDNA in the tissue tested relative to that in the tail of the same individual at day 0 after birth. Data are presented as mean ± SD. Asterisks indicate significant differences (*P* < 0.05). NS, not significant. (B) Dynamics of the proportion of ∆mtDNA in peripheral blood samples from single individuals during early life. Red and blue symbols indicate cases where the proportion of ∆mtDNA in peripheral blood samples increased and decreased, respectively, between days 14 and 34 after birth. Asterisk indicates significant differences (*P* < 0.05). (C) Cytological observations of peripheral blood samples obtained from mito-mice∆ at day 34 after birth. Peripheral blood samples carrying 0%, 38%, and 59% ∆mtDNA, respectively, were stained with new methylene blue and reticulocytes (open arrowheads) are visualized similar to Figure 3D. Scale bar, 10 μm. (D) Average proportion of reticulocytes in total RBC. Results for peripheral blood samples obtained from mito-mice∆ carrying 0%, 7–38%, and 53–64% ∆mtDNA are shown as white, gray, and black colors, respectively. Data are presented as mean ± SD. NS, not significant. (E) Histologic observations of iron metabolism in liver samples from mito-mice∆ at day 34 after birth. Liver samples carrying 0%, 38%, and 47% ∆mtDNA, respectively, were stained with Prussian blue. Scale bar, 10 μm. (F) COX-EM of liver samples obtained from mito-mice∆ at day 34 after birth. The COX-EM of liver samples carrying 0%, 25%, and 47% ∆mtDNA, respectively, are shown. The loading of 47% ∆mtDNA was the maximum in the liver samples that we examined at day 34, because the proportion of ∆mtDNA in liver was lower than that in tissues during early life. N, nucleus. Bar, 2 µm.

The results of the early life span assay described previously (see Figure 1E) clearly showed that there were two populations of neonates carrying ≥50% ∆mtDNA in their tail; the first population showed death phenotypes in the first month after birth, probably due to PS-like phenotypes; and the second population was able to escape early death. In mito-mice∆, genetic variation is restricted to the load of ∆mtDNA in various tissues, so that phenotypes are strictly induced by the load of ∆mtDNA in affected tissues. Considering the point, there was a possibility that that the ∆mtDNA load decreased preferentially in the peripheral blood and liver of surviving neonates by around 1-month after birth. To test this possibility, we examined ∆mtDNA load in affected and nonaffected tissues of surviving neonates at various time points up to and including day 34 after birth (Figure 4A). In this assay, we could not trace the proportion of ∆mtDNA load in tissues from single neonates, because we had to kill neonates to obtain tissue samples. Thus, we estimated the proportion of ∆mtDNA in each sample relative to that in the tail sample taken on day 0 after birth from the same subject. The animals were analyzed as two subgroups defined by the ∆mtDNA load in the tail (*i.e.*, <50% and ≥50% ∆mtDNA). In kidney samples as a nonaffected tissue, the relative ratios increased with time in both subgroups, suggesting that the proportion of ∆mtDNA in the nonaffected tissues increased independently of the ∆mtDNA load (Figure 4A, *P* < 0.05). In peripheral blood, there were two distinct patterns dependent on ∆mtDNA load. In neonates carrying <50% ∆mtDNA in their tails at day 0, the relative ratios increased with time and the pattern was similar to that of kidney samples (*P* < 0.05). In neonates carrying ≥50% ∆mtDNA in their tails at day 0, the relative ratios in peripheral blood samples did not increase with time. The relative ratios in liver samples from both subgroups did not increase with time. In the case of liver samples from E18.5 embryos carrying ≥50% ∆mtDNA, however, a significant decrease of relative ratios was observed (*P* < 0.05).

To confirm changes in ∆mtDNA load in peripheral blood within same individuals, we collected peripheral blood samples from single neonates carrying 7–64% ∆mtDNA, respectively, in their tails at day 0. We calculated the proportion of ∆mtDNA in peripheral blood on day 34 after birth relative to that on day 14 after birth (Figure 4B). The relative ratios in surviving neonates carrying <50% ∆mtDNA in their tails were >1 and particularly high in neonates carrying <20% ∆mtDNA in their tails. In contrast, neonates carrying ≥50% ∆mtDNA in their tails showed relative ratios <1. There was a significant difference between neonates carrying <50% and ≥50% ∆mtDNA in their tails (*P* < 0.05). These data indicate that the proportion of ∆mtDNA in peripheral blood decreased at an early age (*i.e.*, from days 14 to 34) when the proportion of ∆mtDNA in peripheral blood was ≥50%.

Consistent with these results, abnormalities observed in peripheral blood and liver samples of late-stage embryos (Figure 3, D–G) were not detected on day 34 after birth (Figure 4, C–F). There was no significant difference in reticulocyte number between wild-type mice and mito-mice∆ (Figure 4, C and D). Iron deposits that were seen in livers from late stage embryos carrying ≥50% ∆mtDNA were not observed in the livers carrying 47% ∆mtDNA, which was the maximum proportion of ∆mtDNA observed on day 34 after birth (Figure 4E) because of the decreased ∆mtDNA loads in liver. In COX-EM, abnormalities were not observed in liver samples (Figure 4F). These findings indicate that the decrease of ∆mtDNA loading proportion in peripheral blood and liver of neonates carrying ≥50% ∆mtDNA would be one reason for recovering from PS-like phenotypes and for the resultant survival.

### Disease phenotypes of surviving neonates when they reach middle life

**Figure 5:**
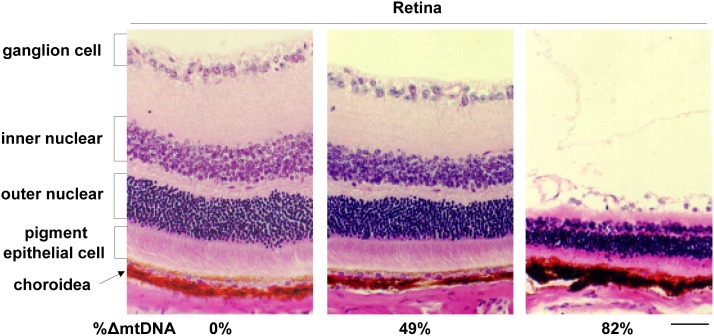
Phenotypic observations of middle-aged mito-mice∆. Histological observations of retina from mito-mice∆ assayed at 6 months of age. Sections of eye samples were stained with hematoxylin and eosin. The proportion of ∆mtDNA in eye samples from the opposite side is indicated under the each picture. Scale bar, 50 µm.

At 6 months of age or later, all the surviving mito-mice∆ showed lactic acidosis, heart block, renal failure, male infertility, deafness, and abnormalities in long-term memory as reported previously, when the proportion of ∆mtDNA in affected tissues reached approximately 70–80% (Inoue *et al.* 2000; Nakada *et al.* 2001, 2004, 2006; Ogasawara *et al.* 2010; Tanaka *et al.* 2008). In some of surviving mito-mice∆ carrying >80% ∆mtDNA, retinal abnormalities were observed (Figure 5). These results indicated that neonates that remitted from PS-like phenotypes in early life expressed KSS-like phenotypes in their middle life.

## Discussion

**Figure 6:**
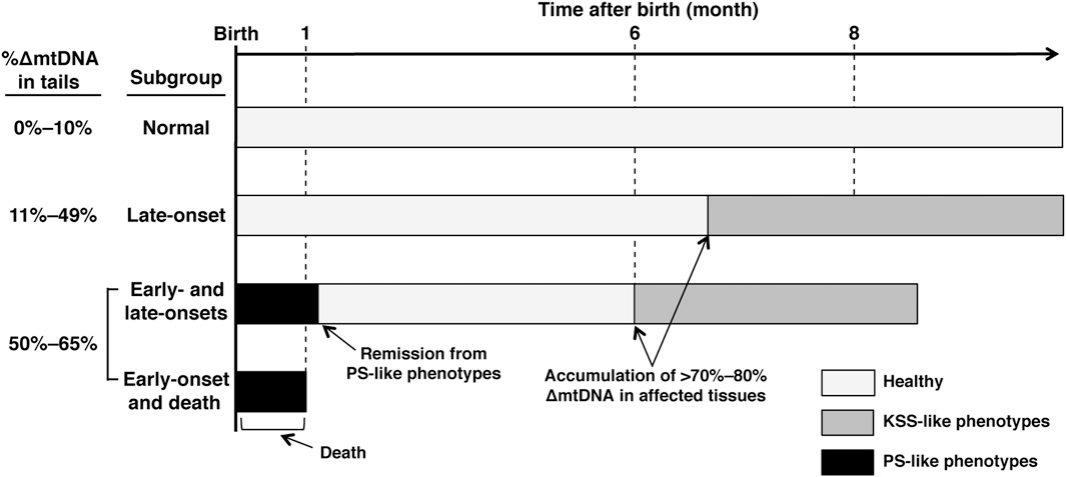
Schematic representation of disease phenotypes in mito-mice∆ population. “Normal” and “late-onset” subgroups in mito-mice∆ population and their clinical features have already been reported as our previous studies (Inoue *et al.* 2000; Nakada *et al.* 2001, 2004, 2006; Ogasawara *et al.* 2010; Tanaka *et al.* 2008). The novelty of this study was to found out two subgroups, “early-onset and death” and “early- and late-onsets” in addition of conventional two subgroups, “normal” and “late-onset” subgroups.

Taking the current results together with our previous ones, we summarized the development of mitochondrial diseases in mito-mice∆ (Figure 6). On the basis of the difference in ∆mtDNA loads and the resultant mitochondrial disease phenotypes, mito-mice∆ population is classified into four subgroups. The first subgroup was mito-mice∆ carrying ≤10% ∆mtDNA at the birth. They showed normal phenotypes because ∆mtDNA loads did not reach >70–80% in various tissues through the life (Normal subgroup in Figure 6). The second subgroup was mito-mice∆ carrying 11–49% ∆mtDNA just after birth, and the mice appeared healthy until middle age (6 months) or later, but when the ∆mtDNA load reached >70–80% in various tissues, they expressed KSS-like phenotypes (late-onset subgroup in Figure 6). The main clinical features in this subgroup, such as low body weight, lactic acidosis, heart block, renal failure, deafness, male infertility, and abnormal long-term memory, has been reported previously (Inoue *et al.* 2000; Nakada *et al.* 2001, 2004, 2006; Ogasawara *et al.* 2010; Tanaka *et al.* 2008). The third and fourth subgroups were mito-mice∆ carrying ≥50% ∆mtDNA just after birth. When the ∆mtDNA load in late-stage embryos reached ≥50%, abnormalities of hematopoiesis and iron metabolisms in liver were induced, thus leading to PS-like phenotypes (*i.e.*, abnormalities in hematopoiesis and iron metabolism; Figure 3, D–F), although >70–80% ∆mtDNA loads were necessary for the onset of KSS-like phenotypes in adult mito-mice∆. Half of those expressing PS-like phenotypes died in early life (≤34 days after birth). The third subgroup, therefore, was the case of onset of PS-like phenotypes and the resultant early death (early-onset and death subgroup in Figure 6). The rest escaped from the early death, because PS-like phenotypes in them disappeared consistent with a decrease of ∆mtDNA load in affected tissues (see Figure 4, D and E for the cases of peripheral blood and liver samples, respectively). However, they expressed KSS-like phenotypes, when ∆mtDNA load reached >70–80% in affected tissues (Figure 5). Thus, the fourth subgroup was the case of transient onsets of PS-like and KSS-like phenotypes in early and middle age, respectively, in a single mito-mice∆ (Early-and late-onsets subgroup in Figure 6).

Heterogeneous distribution of mitochondria with and without COX activity in single cells would be important for elucidating the reason why the pathogenic threshold load of ∆mtDNA in embryonic blood and liver was lower than that in other tissues. We have previously reported that a uniform distribution of mitochondria either with or without COX activity occurs in somatic cells of adult mito-mice∆, irrespective of whether the tissues contain low or high loads of ∆mtDNA (Nakada *et al.* 2001). This finding indicates the occurrence of mitochondrial genetic complementation in which individual mitochondria exchange gene products derived from mtDNA through their fusion and fission. Because mitochondria carrying ∆mtDNA can be supplied by mitochondria carrying wild-type mDNA, tissues carrying ∆mtDNA did not show mitochondrial respiration defects until ∆mtDNA accumulated to a level of >70–80%. In contrast, here we observed that blood cells and hepatocytes in late-stage embryos showing PS-like phenotypes contained mitochondria with heterogeneous ultra-structures and COX activity levels (Figure 3, G and H). This finding suggested the existence of deficiencies of mitochondrial fusion or fission or both, and a lack of mitochondrial genetic complementation in these late-stage embryonic cells. Such deficiencies could be one reason that a load of ≥50% ∆mtDNA could cause pathogenicity in blood cells and hepatocytes of late-stage embryos, whereas a higher load is required in adult mice. Moreover, it has been reported that mitochondrial dynamics, a continuous mitochondrial fusion and fission, is necessary to maintain normal mitochondrial respiratory function in mammals (Chen *et al.* 2010; Ishihara *et al.* 2009). Therefore, it is possible that deficiencies of mitochondrial dynamics and genetic complementation in embryonic blood cells and hepatocytes enhance the pathogenicity of ∆mtDNA in the case of a ≥50% ∆mtDNA load.

PS-like phenotypes induced by the abnormalities of hematopoiesis and iron metabolisms in livers of late-stage embryos carrying ≥50% ∆mtDNA were the main clinical features, but it is unclear why a half of the neonates carrying ≥50% ∆mtDNA died early. There are several possible explanations for this phenomenon. The first is that the pups might have suffered acute oxidative stress because newborns and infants are particularly prone to oxidative stress (Saugstad 2005). Newborns and infants have reduced antioxidant defense mechanisms as well as high levels of free iron, which are required for the Fenton reaction (Saugstad 2005); approximately 20% neonates died on day 1 after birth even in wild-type B6 mice (Figure 1, B and C). Because neonates carrying ≥50% ∆mtDNA in their tails possessed iron deposits in liver (Figure 3F), the high level of iron could promote the Fenton reaction, resulting in additional oxidative stress when the neonates are exposed to a high oxygen concentration after delivery (Singer and Muhlfeld 2007). Another possibility was that abnormalities in metabolic adaptation to the aerobic condition just after birth might cause frequent early death in mito-mice∆. Anaerobic glycolysis is the major source of cellular ATP in fetal tissues. In cells with mitochondrial respiration defects as the result of pathogenic mutant mtDNAs, glycolysis is enhanced as a compensatory mechanism to maintain ATP levels. Therefore, ∆mtDNA might not demonstrate its pathogenicity in various cells during embryogenesis. Supporting this notion, we observed only rare incidents of embryonic lethality in mito-mice∆. After birth, neonates carrying ∆mtDNA have to change from anaerobic to aerobic energy metabolism in various cells as soon as possible, but they would find this process difficult due to their systemic and chronic mitochondrial respiration defects.

In summary, our model mouse study showed that a single ∆mtDNA molecule has a pathogenic potential to cause PS-like and KSS-like phenotypes at early and middle life, respectively, and also suggested that difference in threshold for tolerance to accumulation of ∆mtDNA in affected tissues between young infants and adults was a possible reason for the onset of disease phenotypes in mito-mice∆. In addition, dynamics of ∆mtDNA load in affected tissues well correlated with the transition of disease phenotypes in mito-mice∆. At this sage, we considered that decreased proportion of ∆mtDNA in peripheral blood and liver from young infants carrying >50% ∆mtDNA was important to recover from PS-like phenotypes. However, we could not rule out a possibility that the decreased proportion of ∆mtDNA in affected tissues was a result by which young infants carrying >50% ∆mtDNA recovered from PS-like phenotypes. Therefore, biological and clinical significance of ∆mtDNA dynamics associating with changes of disease phenotypes remains to be answered.
